# Heredity and Environment

**Published:** 1916-10

**Authors:** G. Henri Bogart

**Affiliations:** Shelbyville, Ill.


### Heredity and Environment.

BY DR. G. HENRI BOGART, SHELBYVILLE, ILL.

We hear a great deal about the influence of heredity, and equally strong appeals are urged for the power of environment. Each of these influences have supporters who claim paramount good or ill for his own side.

How would it do to consider these two influences as equal factors, one yielding support to the other and each equally determining.

Concrete illustrations have always taught me more than naked facts, so I shall apply such thought to the question.

Heredity is the warp, the thread of life fixed from end to end of the web which shall be woven in a life. It is fixed, final, fateful from birth unto death. Environment is the woof of the same web. The cunning weaver takes the loom and sends the shining shuttle back and forth, back and forth, and at his will the web may be woven into coarse buckram, or it may come forth in damascene figures of gorgeous beauty. Both warp and woof are needful, there will be no cloth with either lacking.

Agassiz revolutioned American science by remarking a lecture which he was to discuss: "It is true to fact, it pictures the facts with marvelous fidelity, but it is not at all comparative, and lacking comparison, it lacks all but bare assertion, it is valueless."

To deny the force of environment would be to deny the progress of evolution. We are growing, the race is growing, ever growing.

The wise ones, the Marconis and the Edisons are standing on the outer edge of the plane of human achievement, and reaching from the known, they grasp into the related unknown, and show us, that we, too, may know, thus ever widening the range of our inception and conception of the universe and its secrets, for the Allgood gives to us only the knowledge which we win.

Environments long continued, through successive generations, become heredities. It is this seizing upon certain results of environment which have made Burbank a wizard, only his wizardry is merely patient and loving observation and that infinite patience and persistency which is genius, and which aids in unfolding the bud into the flower, a bit the sooner.

There must be a basis of heredity, on which to put the spell of environmental development, and the rough, unlovely threads of an adverse heredity must be skillfully hidden and covered by the woof of the life web, and the coarser and the rougher the foundation, the greater must be the skill and the kindliness of the weaver, to give a beautiful fabric.

To agree that heredity is final as well as fateful and fixed is to refuse the entire ideal of growth or betterment; it is the doctrine of the pessimistic fatalist, while to refuse the tremendous power of heredity is to meet failure, foredoomed. Once, we of the medical profession, talked of hereditary tuberculosis, and now we know that it was but a diathesis which the unfortunate one inherited, and that better environment, made specially good, will overcome the bad inheritance.

I would not wish to live in a world, from which the rainbow of Hope were banished, even the Greek myth left that one blessing to the race, when Pandora's curiosity opend the fateful box. But on the other hand, one should exercise care and caution in trusting alone to hope and aspiration, for facts are stubborn and will assert themselves. As ever, the middle course is the only safe way, and the wise physician is he who realizes that both heredity and environment are equally potent, and who seeks to eliminate the baleful and to help the desirable in mind, soul, body or conditions to betterment, that the good shall grow and the evil lessen from the world.

---

Dr. Malone Duggan of San Antonio, who is a member of the Medical Reserve Corps, was ordered to duty July 27, and is now in service in the base hospital, Brownsville, Texas.

